# L-PGDS-produced PGD_2_ in premature, but not in mature, adipocytes increases obesity and insulin resistance

**DOI:** 10.1038/s41598-018-38453-y

**Published:** 2019-02-13

**Authors:** Ko Fujimori, Kosuke Aritake, Yo Oishi, Nanae Nagata, Toko Maehara, Michael Lazarus, Yoshihiro Urade

**Affiliations:** 1 Department of Pathobiochemistry, Osaka University of Pharmaceutical Sciences, 4-20-1 Nasahara, Takatsuki, Osaka 569-1094 Japan; 2 Laboratory of Chemical Pharmacology, Daiichi University of Pharmacy, 22-1 Tamagawa-cho, Minami-ku, Fukuoka 815-8511 Japan; 3 International Institute for Integrative Sleep Medicine (WPI-IIIS), University of Tsukuba, 1-1-1 Tennodai, Tsukuba, Ibaraki 305-8575 Japan; 4 Department of Animal Radiology and Graduate School of Agriculture and Life Sciences, The University of Tokyo, 1-1-1 Yayoi, Bunkyo-ku, Tokyo 113-8657 Japan; 5 The University of Tokyo Hospital, 7-3-1 Hongo, Bunkyo-ku, Tokyo 113-8655 Japan; 6 Graduate School of Pharmaceutical Sciences, Kitasato University, 5-9-1 Shirokane, Minato-ku, Tokyo 108-8641 Japan

## Abstract

Lipocalin-type prostaglandin (PG) D synthase (L-PGDS) is responsible for the production of PGD_2_ in adipocytes and is selectively induced by a high-fat diet (HFD) in adipose tissue. In this study, we investigated the effects of HFD on obesity and insulin resistance in two distinct types of adipose-specific L-PGDS gene knockout (KO) mice: *fatty acid binding protein 4* (*fabp4*, *aP2*)*-Cre/L-PGDS* ^*flox/flox*^ and *adiponectin* (*AdipoQ*)*-Cre/L-PGDS* ^*flox/flox*^ mice. The L-PGDS gene was deleted in adipocytes in the premature stage of the former strain and after maturation of the latter strain. The L-PGDS expression and PGD_2_ production levels decreased in white adipose tissue (WAT) under HFD conditions only in the *aP2-Cre/L-PGDS* ^*flox/flox*^ mice, but were unchanged in the *AdipoQ-Cre/L-PGDS* ^*flox/flox*^ mice. When fed an HFD, *aP2-Cre/L-PGDS* ^*flox/flox*^ mice significantly reduced body weight gain, adipocyte size, and serum cholesterol and triglyceride levels. In WAT of the HFD-fed *aP2-Cre/L-PGDS* ^*flox/flox*^ mice, the expression levels of the adipogenic, lipogenic, and M1 macrophage marker genes were decreased, whereas those of the lipolytic and M2 macrophage marker genes were enhanced or unchanged. Insulin sensitivity was improved in the HFD-fed *aP2-Cre/L-PGDS* ^*flox/flox*^ mice. These results indicate that PGD_2_ produced by L-PGDS in premature adipocytes is involved in the regulation of body weight gain and insulin resistance under nutrient-dense conditions.

## Introduction

Obesity is a critical health problem worldwide and is now reaching pandemic levels^1^. Obesity occurs due to an imbalance between energy intake and energy expenditure, and is associated with various health problems including type 2 diabetes, atherosclerosis, hypertension, and cardiovascular diseases^2,3^. Adipose cells are a major energy storage site for lipids in mammals, and are involved in the control of energy homeostasis^4^. Moreover, adipose tissue has been identified as the endocrine organ that secretes a variety of adipocytokines^5^.

Adipocyte differentiation (adipogenesis) occurs via the multiple and complex processes. Transcription regulatory mechanism in adipocyte differentiation has been extensively studied, and a number of transcription factors involved in this regulation have been identified. Among them, CCAAT/enhancer-binding proteins (C/EBPs), peroxisome proliferator-activated receptor (PPAR) γ, and sterol regulatory element-binding protein-1c (SREBP-1c) are critical in the regulation of adipogenesis^6–8^. These transcription factors regulate gene expression for various adipogenic proteins, which are involved in the regulation of adipogenesis^6–8^.

Prostaglandins (PGs) are members of the lipid mediators, some of which are involved in the regulation (activation or suppression) of adipogenesis^9,10^. PGD_2_ enhances the progression of adipogenesis^11^, and its metabolites, 15-deoxy-Δ^12,14^-PGJ_2_ (15d-PGJ_2_)^12,13^ and Δ^12^-PGJ_2_^14^ activate adipogenesis via a nuclear receptor, PPARγ. In contrast, PGE_2_ and PGF_2α_ are involved in the suppression of adipogenesis. PGE_2_ is produced by microsomal PGE synthase-1 in adipocytes^15^ and represses adipogenesis through the EP4 receptors^16^ by increasing the synthesis of anti-adipogenic PGE_2_ and PGF_2α_ in mouse embryonic fibroblasts (MEFs)^17^ and mouse adipocytic 3T3-L1 cells^18^. PGF_2α_ is synthesized by aldo-keto reductase 1B3^19^ and 1B7^20^ in adipocytes, and represses the progression of the early stage of adipogenesis via the FP receptors^21–23^.

There are two distinct types of PGD synthase (PGDS). One is lipocalin-type PGDS (L-PGDS) and the other is hematopoietic PGDS (H-PGDS). The L-PGDS gene is highly expressed in the brain, heart, and male genital organs^24^. Whereas H-PGDS is responsible for the synthesis of PGD_2_ in inflammatory and immune cells, i.e., macrophages, mast cells, and Th2 cells^25,26^. PGD_2_ exerts its physiological effects through two G protein-coupled receptors, the PGD_2_ receptors (DP), DP1 receptors and chemoattractant receptor-homologous molecule expressed on Th2 cells (CRTH2), DP2 receptors^27^.

L-PGDS-produced PGD_2_ enhances lipid accumulation in 3T3-L1 cells^11,14^ through suppression of lipolysis via the DP2 receptors^28^. *In vivo* studies have been carried out using the gene-manipulated mice of the L-PGDS gene. PGD_2_-overproducing mice fed a high-fat diet (HFD) became obese^29^. L-PGDS gene knockout (KO) mice showed glucose intolerance and insulin resistance, and increased fat mass in the aorta under HFD conditions^30^. Adipose cells of the L-PGDS KO mice were larger than those of mice fed low-fat diet (LFD)^30^. L-PGDS KO mice showed no change in body weight, but improved glucose tolerance under HFD conditions^31^. In contrast, no glucose or insulin intolerance was observed in L-PGDS KO mice, but body weight gain and atherosclerotic lesions in the aorta were increased^32^. Thus, the roles of L-PGDS in obesity and obesity-related phenotypes in the L-PGDS gene-manipulated mice remain controversial. PGD_2_ is involved in the regulation of various physiological events and L-PGDS is widely expressed in the body^33^. The disruption of the L-PGDS gene throughout the whole body may cause the unexpected effects and/or the unexplained phenotypes.

To address these concerns, we investigated the adipose-specific functions of L-PGDS and PGD_2_ by the use of adipose-specific L-PGDS KO mice under the control of fatty acid binding protein 4 (Fabp4, aP2)-Cre transgene (*aP2-Cre/L-PGDS* ^*flox/flox*^) or adiponectin (AdipoQ)-Cre transgene (*AdipoQ-Cre/L-PGDS* ^*flox/flox*^) through the Cre-loxP system. The *aP2-Cre/L-PGDS* ^*flox/flox*^ mice exhibited decreased body weight gain with the reduction of fat mass, and improved insulin sensitivity under HFD conditions. Therefore, L-PGDS may be a target for the development of anti-obesity medicine and the treatment of obesity-mediated insulin resistance.

## Results

### Expression profile of the L-PGDS gene

**Figure 1:**
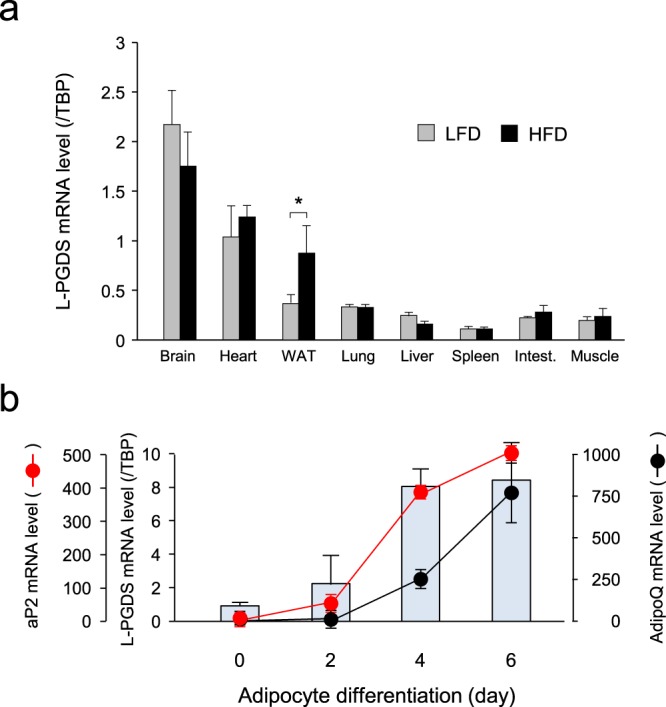
Expression profiles of L-PGDS gene in mice and adipocytes. (**a**) Tissue distribution of L-PGDS gene expression in mice. Mice (C57BL/6N, 5-week-old; n = 3) were fed LFD (*gray columns*) or HFD (*black columns*) for 8 weeks. RNA was prepared from various tissues, and the mRNA levels of the L-PGDS gene were measured by qPCR. Data are shown as means ± S.D. *p < 0.01, as indicated by the brackets. (**b**) Comparison of expression profiles of L-PGDS (*blue columns*), aP2 (*red circles*), and AdipoQ (*black circles*) genes during adipogenesis of mouse 3T3-L1 cells. 3T3-L1 cells were differentiated into adipocytes for 6 days. RNA was prepared and qPCR analysis was performed to measure the transcription levels of the L-PGDS, aP2, and AdipoQ genes. Data are represented as means ± S.D.

For the various tissues of LFD- and HFD-fed mice, the expression of the L-PGDS gene was the highest in the brain, followed by the heart in both LFD- and HFD-fed mice and in white adipose tissue (WAT) in HFD-fed mice (Fig. 1a). The mRNA level of the L-PGDS gene in WAT of HFD-fed mice was selectively enhanced approximately 2.3-fold as compared with LFD and was almost unchanged in other tissues (Fig. 1a).

We then examined the expression of the L-PGDS gene and two adipogenic marker genes, aP2 (Fabp4) and adiponectin (AdipoQ) during adipogenesis of mouse adipocyte 3T3-L1 cells (Fig. 1b). The transcription of the aP2 gene was induced in premature adipocytes even at 2 days after the initiation of adipogenesis and was gradually enhanced during adipogenesis, whose profile closely resembled that of the L-PGDS gene. On the other hand, the expression of the AdipoQ gene was very low at 2 days in premature adipocytes and was induced at 4 days in the mature stage of adipogenesis, indicating that the AdipoQ gene was selectively expressed in mature adipocytes and that its expression came later than those of the L-PGDS and aP2 genes.

### Generation and molecular characterization of adipose-specific L-PGDS KO mice

**Figure 2:**
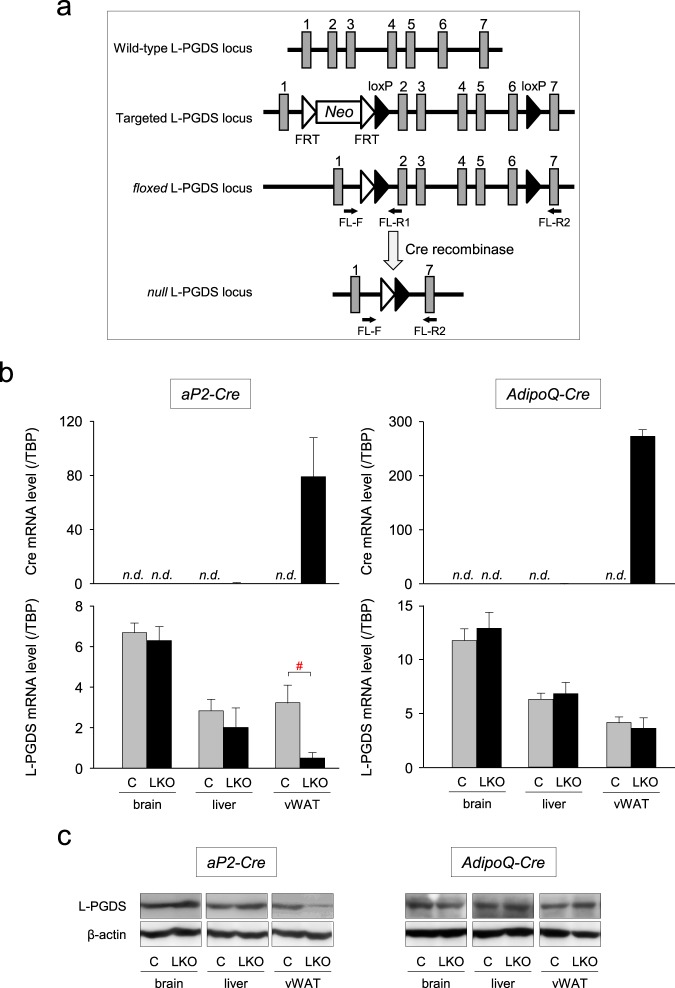
Adipose-specific L-PGDS KO mice. (**a**) Strategy to generate the *aP2-Cre*/*L-PGDS* ^*flox/flox*^ and *AdipoQ-Cre*/*L-PGDS* ^*flox/flox*^ mice. Wild-type, targeted, *floxed*, and *null* L-PGDS gene loci were shown. Primers used for genotyping by PCR were indicated as *arrows*. Exons were shown as *boxes*, and the number of exons were shown above *boxes*. *Neo* means the gene for neomycin phosphotransferase. FRT means the flippase recognition target. (**b**) Expression of the Cre and L-PGDS genes in the HFD-fed wild-type (W; *white columns*), *L-PGDS* ^*flox/flox*^ (C; *gray columns*), *aP2-Cre*/*L-PGDS* ^*flox/flox*^ and *AdipoQ-Cre*/*L-PGDS* ^*flox/flox*^ (LKO; *black columns*) mice. Mice (7-week-old; n = 5) were fed an HFD for 11 weeks and the mRNA levels of both genes in the brain, liver, and vWAT was measured by qPCR. *n.d*. means *not detected*. Data are shown as means ± S.D. ^#^*p* < 0.01, as indicated by the bracket. (**c**) Western blot analysis of L-PGDS expression in the brain, liver, and vWAT of the HFD-fed *L-PGDS* ^*flox/flox*^ (C), *aP2-Cre*/*L-PGDS* ^*flox/flox*^, and *AdipoQ-Cre*/*L-PGDS* ^*flox/flox*^ (LKO). Protein (20 μg) was applied in each lane. Data are the representative of each of the three mice.

To study the roles of L-PGDS and PGD_2_ in obesity, we generated the experimental mice by breeding the *L-PGDS* ^*flox/flox*^ mice with either of two distinct types of adipose-specific L-PGDS gene knockout (KO) mice: *aP2-Cre/L-PGDS* ^*flox/flox*^ and *AdipoQ-Cre/L-PGDS* ^*flox/flox*^ mice (Fig. 2a).

The *null* L-PGDS allele was detected in visceral WAT (vWAT) of the HFD-fed *aP2-Cre/L-PGDS* ^*flox/flox*^ mice, but not in the *AdipoQ-Cre/L-PGDS* ^*flox/flox*^ mice (513-bp), although the *floxed* L-PGDS was detected in all tissues of both *L-PGDS* ^*flox/flox*^ and *aP2-Cre/L-PGDS* ^*flox/flox*^ mice (2513-bp; Supplemental Fig. S1). The body weight gains of wild-type and *L-PGDS* ^*flox/flox*^ mice were almost the same under LFD or HFD conditions (Supplemental Fig. S2a). Moreover, the expression levels of the L-PGDS mRNA in the brain, liver, and vWAT of the *L-PGDS* ^*flox/flox*^ mice were almost the same as those of wild-type mice under LFD or HFD conditions (Supplemental Fig. S2b).

The Cre transgene was abundantly expressed in vWAT under the control of the aP2 promoter/enhancer or AdipoQ promoter, but not in the brain and liver (Fig. 2b). In addition, the mRNA for the Cre transgene was not detected in wild-type mice (Fig. 2b). The mRNA level of the L-PGDS gene was significantly reduced in vWAT of the HFD-fed *aP2-Cre/L-PGDS* ^*flox/flox*^ mice, but unchanged in their brains and livers, as compared with that of the control *L-PGDS* ^*flox/flox*^ mice (Fig. 2b). In contrast, the adipose-specific decrease in the L-PGDS mRNA level was not detected in vWAT of the *AdipoQ-Cre/L-PGDS* ^*flox/flox*^ mice, although the Cre transgene was expressed in WAT in these mice under the control of the AdipoQ promoter (Fig. 2b). Furthermore, to confirm a decrease in L-PGDS protein, we carried out Western blot analysis. The expression of L-PGDS protein in the brain and liver of the HFD-fed *aP2-Cre/L-PGDS* ^*flox/flox*^ or *AdipoQ-Cre/L-PGDS* ^*flox/flox*^ mice was almost the same as those in the *L-PGDS* ^*flox/flox*^ mice (Fig. 2c). In contrast, L-PGDS expression was clearly lowered in vWAT of HFD-fed *aP2-Cre/L-PGDS* ^*flox/flox*^ mice, but not in vWAT of the HFD-fed *AdipoQ-Cre/L-PGDS* ^*flox/flox*^ mice (Fig. 2c).

These results reveal that the HFD-fed *aP2-Cre/L-PGDS* ^*flox/flox*^ mice exhibited the adipose-specific decrease in L-PGDS gene expression.

### Decrease of body weight gain in the HFD-fed *aP2-Cre/L-PGDS*^*flox/flox*^ mice

**Figure 3:**
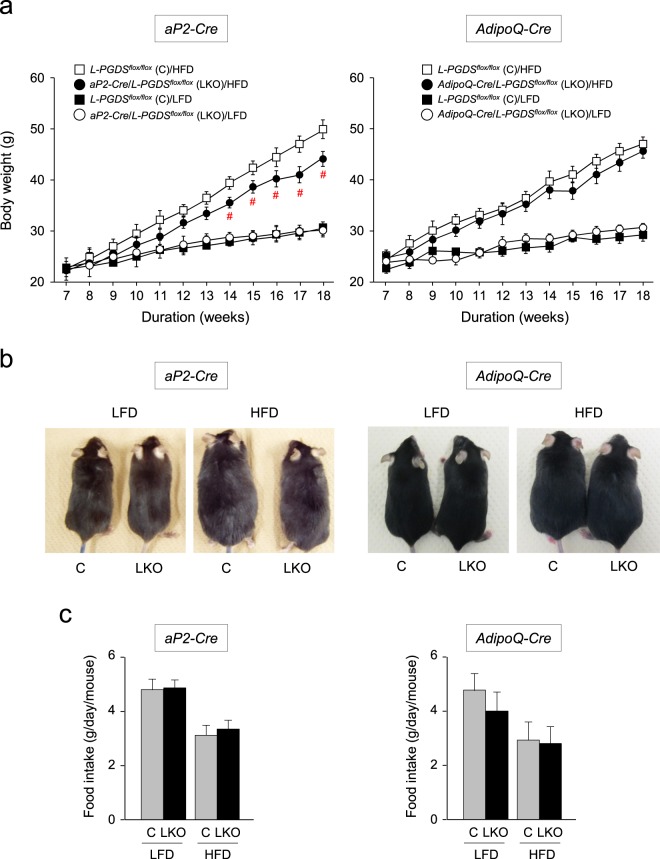
Change of body weight gain in the *aP2-Cre*/*L-PGDS* ^*flox/flox*^ and *AdipoQ-Cre*/*L-PGDS* ^*flox/flox*^ mice. (**a**) Body weight change in the *aP2-Cre*/*L-PGDS* ^*flox/flox*^, *AdipoQ-Cre*/*L-PGDS* ^*flox/flox*^, and control *L-PGDS* ^*flox/flox*^ mice (7-week-old; *n = *5–6) under LFD or HFD conditions for 11 weeks. Body weight was measured every week. Data are representative of 4 independent experiments (n = 4–6) and shown as means ± S.D. ^#^*p* < 0.01, as compared with the HFD-fed *L-PGDS* ^*flox/flox*^ mice. (**b**) Photographs of the representative 18-week-old control *L-PGDS* ^*flox/flox*^ (C), *aP2-Cre*/*L-PGDS* ^*flox/flox*^ and *AdipoQ-Cre*/*L-PGDS* ^*flox/flox*^ (LKO) mice under LFD or HFD conditions. (**c**) Daily food intake per gram body weight. Data are shown as means ± S.D. (n = 4–6).

We then measured body weight gains in the *aP2-Cre/L-PGDS* ^*flox/flox*^, *AdipoQ-Cre/L-PGDS* ^*flox/flox*^, and control *L-PGDS* ^*flox/flox*^ mice (7-week-old; n = 5) following 11 weeks of either LFD or HFD. At the start of the experiment, average body weight was not significantly different among the *aP2-Cre/L-PGDS* ^*flox/flox*^, *AdipoQ-Cre/L-PGDS* ^*flox/flox*^, and control *L-PGDS* ^*flox/flox*^ mice (Fig. 3a). Under LFD conditions for 11 weeks, no significant changes in body weight gain were found among the *aP2-Cre/L-PGDS* ^*flox/flox*^, *AdipoQ-Cre/L-PGDS* ^*flox/flox*^, or control *L-PGDS* ^*flox/flox*^ mice (Fig. 3a,b). In contrast, the *aP2-Cre/L-PGDS* ^*flox/flox*^ mice fed an HFD gained significantly less body weight, as compared with those in the control *L-PGDS* ^*flox/flox*^ mice (Fig. 3a,b). On the other hand, body weight gain was not altered in the *AdipoQ-Cre/L-PGDS* ^*flox/flox*^ mice even under HFD conditions, as compared with those of the control *L-PGDS* ^*flox/flox*^ mice (Fig. 3a,b). Food intake rates were similar between *L-PGDS* ^*flox/flox*^ (control) and either *aP2-Cre/L-PGDS* ^*flox/flox*^ or *AdipoQ-Cre/L-PGDS* ^*flox/flox*^ mice under both LFD and HFD conditions (Fig. 3c).

**Figure 4:**
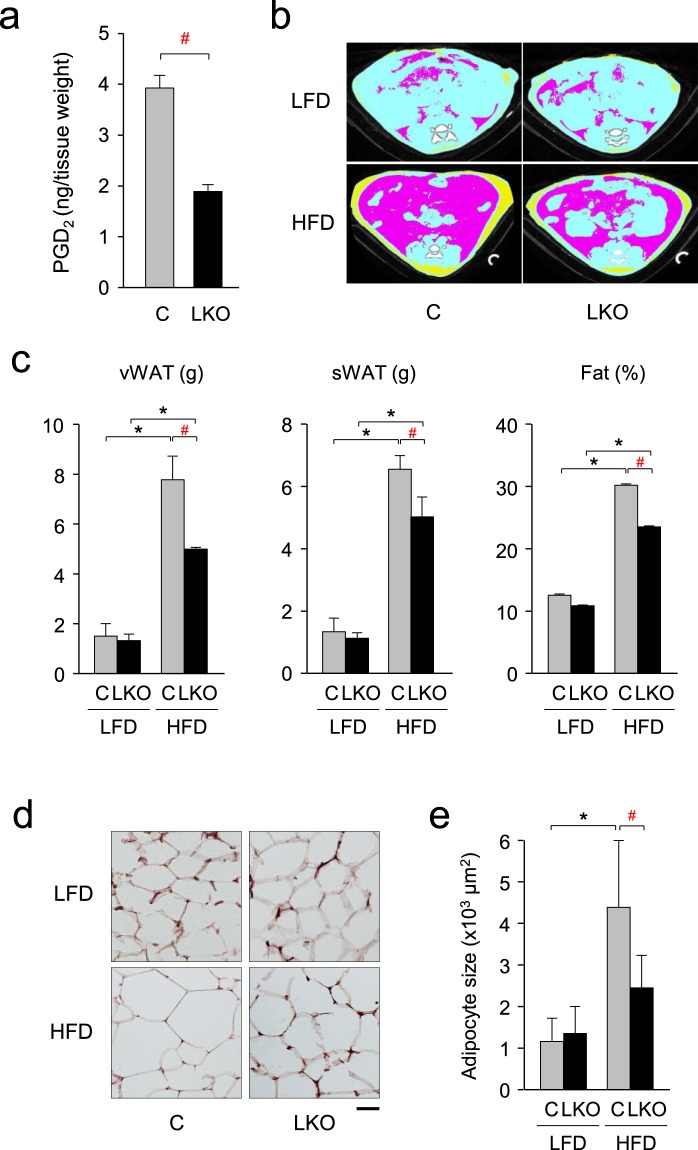
Decrease of body fat in the HFD-fed aP2-Cre/L-*PGDS* ^*flox/flox*^ mice. (**a**) PGD_2_ level in vWAT of the HFD-fed *L-PGDS* ^*flox/flox*^ (C; *gray column*) and *aP2-Cre*/*L-PGDS* ^*flox/flox*^ (LKO; *black column*) mice. Data are shown as means ± S.D. ^#^*p* < 0.01, as indicated by the bracket. (**b**) CT analysis. Abdominal cross-section of the LFD- or HFD-fed control *L-PGDS* ^*flox/flox*^ (C) and *aP2-Cre*/*L-PGDS* ^*flox/flox*^ (LKO) mice (18-week-old). vWAT, sWAT, and internal organs were shown by *pink*, *yellow*, and *light blue*, respectively. (**c**) Quantification of fat (vWAT and sWAT) in the whole body of the LFD- or HFD-fed control *L-PGDS* ^*flox/flox*^ (C) and *aP2-Cre*/*L-PGDS* ^*flox/flox*^ (LKO) mice (18-week-old) by using of LaTheta software (Aloka). Fat (%) means the ratio of the weights of total fat per body weight. Data are shown as means ± S.D. *p < 0.01, as indicated by the brackets. Significant difference between *aP2-Cre*/*L-PGDS* ^*flox/flox*^ (LKO) mice and control *L-PGDS* ^*flox/flox*^ (C) under HFD conditions was shown as ^#^*p* < 0.01 with the brackets. (**d**) Adipose size of vWAT in the 18-week-old control *L-PGDS* ^*flox/flox*^ (C) and *aP2-Cre*/*L-PGDS* ^*flox/flox*^ (LKO) mice under LFD or HFD conditions, stained with hematoxylin and eosin. Bar = 50 μm. (**e**) Quantification of adipocyte area shown in (**d**) was performed on hematoxylin and eosin-stained sections using ImageJ software. At least 200 adipocytes from five mice in each group were measured. Data are shown as means ± S.D. *p < 0.01, as indicated by the brackets. Significant difference between *aP2-Cre*/*L-PGDS* ^*flox/flox*^ (LKO) mice and control *L-PGDS* ^*flox/flox*^ (C) under HFD conditions was shown as ^#^*p* < 0.01 with the brackets.

We further characterized the phenotype of the *aP2-Cre/L-PGDS* ^*flox/flox*^ mice under HFD conditions. PGD_2_ level was lowered in vWAT of the HFD-fed *aP2-Cre/L-PGDS* ^*flox/flox*^ mice to be about 53% of that of the control *L-PGDS* ^*flox/flox*^ mice (Fig. 4a). Computed tomography (CT) analysis (Fig. 4b) revealed that the weights of vWAT and subcutaneous WAT (sWAT) were decreased in the HFD-fed *aP2-Cre/L-PGDS* ^*flox/flox*^ mice to be about 64% and 73%, respectively, of those of the control *L-PGDS* ^*flox/flox*^ mice (Fig. 4c). Moreover, the ratio of fat in body mass {Fat (%)} was reduced in the HFD-fed *aP2-Cre/L-PGDS* ^*flox/flox*^ mice under HFD conditions to be approximately 73% of those of the control *L-PGDS* ^*flox/flox*^ mice (Fig. 4c). While, the body fat mass (vWAT and sWAT) and the ratio of fat in body mass {Fat (%)} were similar between *L-PGDS* ^*flox/flox*^ and *aP2-Cre/L-PGDS* ^*flox/flox*^ mice under LFD conditions (Fig. 4c).

Histological analysis showed that HFD increased adipocyte size approximately 3.9-fold in the *L-PGDS* ^*flox/flox*^ mice, but only about 1.8-fold in the *aP2-Cre/L-PGDS* ^*flox/flox*^ mice (Fig. 4d,e). These results indicate that body weight gain and fat mass increases by HFD were reduced in the *aP2-Cre/L-PGDS* ^*flox/flox*^ mice.

### Change in the expression level of the genes involved in the PG synthetic pathway and PG receptors in vWAT and sWAT of the *aP2-Cre/L-PGDS*^*flox/flox*^ mice

**Figure 5:**
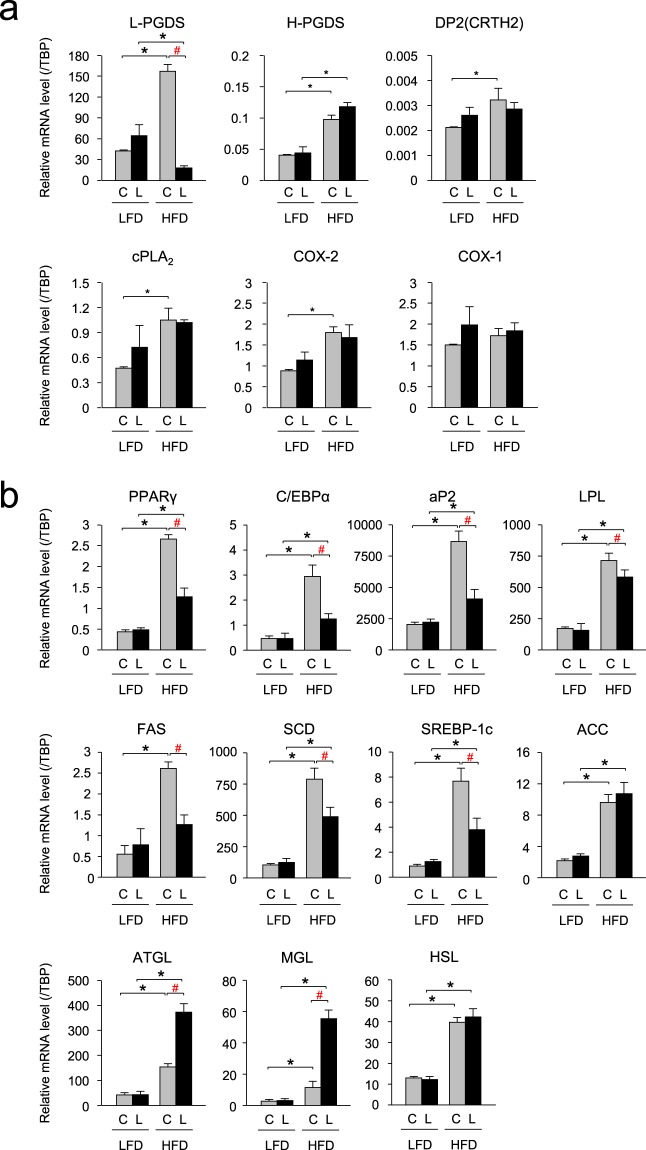
Changes of gene expression levels in vWAT of the LFD- or HFD-fed mice. (**a**) Expression of the PG synthetic genes in vWAT of the LFD- or HFD-fed control *L-PGDS* ^*flox/flox*^ (C: *gray columns*) and *aP2-Cre*/*L-PGDS* ^*flox/flox*^ mice (L: *black columns*; 18-week-old; n = 5–6) under LFD or HFD. The expression levels of the indicated genes were measured by qPCR. Data are present as means ± S.D. *p < 0.01, as indicated by the brackets. Significant difference between *aP2-Cre*/*L-PGDS* ^*flox/flox*^ (LKO) mice and control *L-PGDS* ^*flox/flox*^ (C) under HFD conditions was shown as ^#^*p* < 0.01 with the brackets. (**b**) Expression of the adipogenic, lipogenic, and lipolytic genes in vWAT of LFD- or HFD-fed control *L-PGDS* ^*flox/flox*^ (C: *gray columns*) and *aP2-Cre*/*L-PGDS* ^*flox/flox*^ mice (L: *black columns*; 18-week-old; n = 5–6) under LFD or HFD conditions. The expression levels of the indicated genes were measured by qPCR. Data are present as means ± S.D. *p < 0.01, as indicated by the brackets. Significant difference between *aP2-Cre*/*L-PGDS* ^*flox/flox*^ (LKO) mice and control *L-PGDS* ^*flox/flox*^ (C) under HFD conditions was shown as ^#^*p* < 0.01 with the brackets.

In the *L-PGDS* ^*flox/flox*^ (control) mice, HFD increased the transcription levels of the L-PGDS gene about 3.5- and 1.8-fold in vWAT (Fig. 5a) and sWAT (Supplemental Fig. S3a), respectively, as compared with those in LFD-fed condition. In contrast, in the *aP2-Cre/L-PGDS* ^*flox/flox*^ mice, HFD decreased the L-PGDS mRNA levels to approximately 13% and 34% in vWAT (Fig. 5a) and sWAT (Supplemental Fig. S3a), respectively, of those of the *L-PGDS* ^*flox/flox*^ mice.

In addition, HFD also elevated gene expression of H-PGDS, another PGDS; cPLA_2_, and COX-2, both are upstream enzymes in PGD_2_ biosynthesis; and DP2 (CRTH2) receptors; one of PGD_2_ receptors, about 2.3- and 1.8-, 2.3- and 1.4-, 2.1- and 2.0-, and 1.5-fold, respectively, but unchanged the expression level of the COX-1 gene in vWAT (Fig. 5a) and sWAT (Supplemental Fig. S3a), as compared with those of the control *L-PGDS* ^*flox/flox*^ mice under LFD conditions. However, in the *aP2-Cre/L-PGDS* ^*flox/flox*^ mice, the mRNA levels of those genes were not changed in vWAT (Fig. 5a) and sWAT (Supplemental Fig. S3a). These results indicate that the mRNA levels of the PGD_2_ synthetic genes, except for the L-PGDS gene were not changed in WAT of the *aP2-Cre/L-PGDS* ^*flox/flox*^ mice under HFD conditions.

### Decreased expression of the adipogenic and lipogenic genes in the HFD-fed *aP2-Cre/L-PGDS*^*flox/flox*^ mice

Next, we investigated the expression levels of the adipogenic genes in vWAT and sWAT of the LFD- or HFD-fed *aP2-Cre/L-PGDS* ^*flox/flox*^ and control *L-PGDS* ^*flox/flox*^ mice using qPCR. Under LFD conditions, the transcription levels of the adipogenic genes such as PPARγ, C/EBPα, aP2, and lipoprotein lipase (LPL) were similar between *L-PGDS* ^*flox/flox*^ and *aP2-Cre/L-PGDS* ^*flox/flox*^ mice (Fig. 5b and Supplemental Fig. S3a). HFD increased the mRNA levels of the PPARγ, C/EBPα, aP2, and LPL genes, approximately 6.2- and 5.2-, 5.6- and 4.6-, 4.3- and 2.0-, and 4.2- and 1.6-fold, respectively in vWAT (Fig. 5b) and sWAT (Supplemental Fig. S3a) of the *L-PGDS* ^*flox/flox*^ mice, and about 2.6- and 3.0-, 2.4- and 3.3-, 1.8- and 2.1-, and 3.7- and 1.2-fold, respectively in vWAT (Fig. 5b) and sWAT (Supplemental Fig. S3a) of the *aP2-Cre/L-PGDS* ^*flox/flox*^ mice. The HFD-induced increases in the mRNA levels of these adipogenic genes were clearly lower in the *aP2-Cre/L-PGDS* ^*flox/flox*^ mice than the control *L-PGDS* ^*flox/flox*^ mice. The transcription levels of these genes in HFD-fed *aP2-Cre/L-PGDS* ^*flox/flox*^ mice were decreased about 51% and 37%, 56% and 50%, 53% and 28%, and 18% and 42%, respectively, in vWAT (Fig. 5b) and sWAT (Supplemental Fig. S3b), as compared with each of the *L-PGDS* ^*flox/flox*^ mice.

The mRNA levels of the lipogenic genes: e.g., fatty acid synthase (FAS), stearoyl-CoA desaturase (SCD), and SREBP-1c were mostly unchanged in both vWAT and sWAT between *L-PGDS* ^*flox/flox*^ and *aP2-Cre/L-PGDS* ^*flox/flox*^ mice under LFD conditions (Fig. 5b and Supplemental Fig. S3b). In contrast, HFD enhanced the mRNA levels in vWAT and sWAT of the FAS, SCD, and SREBP-1c genes approximately 4.3- and 3.4-, 7.3- and 6.0-, and 8.1- and 5.2-fold, respectively, of the *L-PGDS* ^*flox/flox*^ mice, and about 1.8- and 2.3-, 3.7- and 2.6-, and 3.1- and 4.3-fold, respectively, in vWAT and sWAT of the *aP2-Cre/L-PGDS* ^*flox/flox*^ mice (Fig. 5b, Supplemental Fig. S3b). The transcription levels of these genes under HFD in vWAT (Fig. 5b) and sWAT (Supplemental Fig. S3b) of the *aP2-Cre/L-PGDS* ^*flox/flox*^ mice were reduced approximately 51% and 37%, 37% and 57%, 50% and 36%, respectively, of the *L-PGDS* ^*flox/flox*^ mice. HFD also increased the expression levels of the ACC gene in vWAT and sWAT (Fig. 5b and Supplemental Fig. S3b), but its gene expression was not altered between *aP2-Cre/L-PGDS* ^*flox/flox*^ mice and *L-PGDS* ^*flox/flox*^ mice. These results suggest that the expression levels of the adipogenic and lipogenic genes were lowered in WAT of the *aP2-Cre/L-PGDS* ^*flox/flox*^ mice under HFD conditions.

### Effect to lipolysis in the HFD-fed *aP2-Cre/L-PGDS*^*flox/flox*^ mice

We investigated the effects of L-PGDS and PGD_2_ in the lipolysis in WAT of the LFD- or HFD-fed *aP2-Cre/L-PGDS* ^*flox/flox*^ and control *L-PGDS* ^*flox/flox*^ mice. The expression levels of the adipose triacylglyceride (TG) lipase (ATGL), HSL, and monoacylglyceride lipase (MGL) genes involved in TG metabolism (lipolysis) were similar in vWAT and sWAT of the *L-PGDS* ^*flox/flox*^ and *aP2-Cre/L-PGDS* ^*flox/flox*^ mice under LFD conditions (Fig. 5b and Supplemental Fig. S3b). In contrast, the mRNA levels of the ATGL and MGL genes were elevated in HFD-fed mice by approximately 3.2- and 9.4-, and 4.3- and 7.6-fold, respectively, in vWAT (Fig. 5b) and sWAT (Supplemental Fig. S3b) in the *L-PGDS* ^*flox/flox*^ mice. Moreover, the transcription levels of the ATGL and MGL genes in vWAT and sWAT were approximately 2.5- and 1.4-, and 4.8- and 1.5-fold higher, respectively, in the *aP2-Cre/L-PGDS* ^*flox/flox*^ mice than those in the *L-PGDS* ^*flox/flox*^ mice (Fig. 5b and Supplemental Fig. S3b). Whereas, the mRNA levels of the HSL gene in vWAT and sWAT were unchanged in both groups of mice, although HFD induced the transcription levels of this gene when compared with those of the *L-PGDS* ^*flox/flox*^ mice (Fig. 5b and Supplemental Fig. S3b). These results reveal that lipolysis may be enhanced in WAT of the *aP2-Cre/L-PGDS* ^*flox/flox*^ mice under HFD conditions.

### Serum metabolic markers in the *aP2-Cre/L-PGDS*^*flox/flox*^ mice

After LFD- or HFD-feeding for 11 weeks, serum non-esterified fatty acid (NEFA) levels were not significantly altered in the *aP2-Cre/L-PGDS* ^*flox/flox*^ and *L-PGDS* ^*flox/flox*^ mice either by LFD or HFD, although the level was slightly higher in the HFD-fed *aP2-Cre/L-PGDS* ^*flox/flox*^ mice than the HFD-fed *L-PGDS* ^*flox/flox*^ mice (Table 1). Under LFD conditions, serum levels of total cholesterol and total lipid were significantly decreased in the *aP2-Cre/L-PGDS* ^*flox/flox*^ mice (Table 1). Further investigation is needed to understand the reason why these levels were decreased in the LFD-fed *aP2-Cre/L-PGDS* ^*flox/flox*^ mice. Moreover, the levels of total cholesterol, HDL-cholesterol, LDL-cholesterol, glucose, and TG were significantly lower in the *aP2-Cre/L-PGDS* ^*flox/flox*^ mice than in the *L-PGDS* ^*flox/flox*^ mice under HFD conditions (Table 1). These results indicate that serum levels of cholesterols, glucose, and TG were lowered in the HFD-fed *aP2-Cre/L-PGDS*^*flox/flox*^ mice.

**Table 1:** Serum biochemical parameters in *L-PGDS* ^*flox/flox*^ and *aP2-Cre*/*L-PGDS* ^*flox/flox*^ mice.

	LFD		HFD	
	C	LKO	C	LKO
NEFA (μEq/L)	1283.3 ± 66.1	1254.8 ± 97.7	1332.0 ± 74.2	1849.8 ± 301.2
Total cholesterol (mg/dL)	135.0 ± 6.7	*112.8 ± 4.6	210.0 ± 3.9	*106.4 ± 3.9
LDL-cholesterol (mg/dL)	12.6 ± 1.9	9.0 ± 1.3	14.8 ± 2.3	8.2 ± 0.6
HDL-cholesterol (mg/dL)	79.8 ± 8.7	67.8 ± 2.8	87.2 ± 2.3	*57.6 ± 2.3
Total lipid (mg/dL)	451.5 ± 8.7	*369.2 ± 11.5	555.0 ± 13.7	*393.2 ± 22.9
Glucose (mg/dL)	115.8 ± 11.9	105.0 ± 8.9	164.2 ± 17.6	*132.4 ± 14.5
TG (mg/dL)	110.3 ± 6.8	114.8 ± 17.6	161.3 ± 13.3	*78.3 ± 5.6

C and LKO mean control *L-PGDS* ^*flox/flox*^ and *aP2-Cre*/*L-PGDS* ^*flox/flox*^ mice (n = 5), respectively. Data are present as means ± S.E. *p < 0.01, vs. control (C).

### Improvement of inflammation in the HFD-fed *aP2-Cre/L-PGDS*^*flox/flox*^ mice

**Figure 6:**
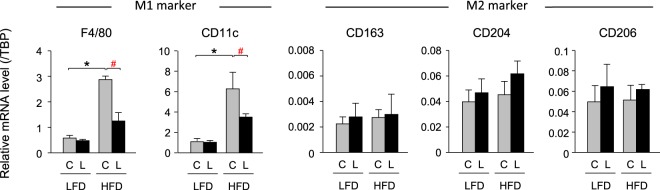
Expression of inflammatory genes in vWAT of LFD- or HFD-fed mice. Expression of the macrophage (M1 and M2) marker genes in vWAT of the LFD- or HFD-fed control *L-PGDS* ^*flox/flox*^ (C: *gray columns*) and *aP2-Cre*/*L-PGDS* ^*flox/flox*^ mice (L: *black columns*; 18-week-old; n = 5–6) under LFD or HFD conditions. The expression levels of the indicated genes were measured by qPCR. Data are present as means ± S.D. *p < 0.01, as indicated by the brackets.

When the expression levels of the macrophage marker genes were measured in vWAT and sWAT of the LFD- and HFD-fed *aP2-Cre/L-PGDS* ^*flox/flox*^ and control *L-PGDS* ^*flox/flox*^ mice, the transcription levels of the M1 macrophage marker genes such as F4/80 and CD11c, were similar in both *aP2-Cre/L-PGDS* ^*flox/flox*^ and *L-PGDS* ^*flox/flox*^ mice under LFD conditions (Fig. 6 and Supplemental Fig. S4). In contrast, HFD elevated the mRNA levels of the F4/80 and CD11c genes in vWAT and sWAT, about 6.3- and 5.0-, and 5.6- and 4.7-fold, respectively, in the *L-PGDS*^*flox/flox*^ mice. However, the mRNA levels of the F4/80 and CD11c genes were not significantly increased in vWAT and sWAT of the *aP2-Cre/L-PGDS* ^*flox/flox*^ mice by HFD feeding (Fig. 6 and Supplemental Fig. S4). The mRNA levels of the F4/80 and CD11c genes were decreased to about 44% and 62%, and 56% and 46%, respectively, in vWAT and sWAT of the *aP2-Cre/L-PGDS* ^*flox/flox*^ mice, as compared with the *L-PGDS* ^*flox/flox*^ mice (Fig. 6 and Supplemental Fig. S4). In contrast, the mRNA levels of the M2 macrophage marker genes; e.g., CD163, CD204, and CD206 were not significantly altered or rather increased in the *aP2-Cre/L-PGDS* ^*flox/flox*^ mice as compared with the *L-PGDS* ^*flox/flox*^ mice in the HFD-fed condition (Fig. 6 and Supplemental Fig. S4). These results suggest that adipose-specific L-PGDS is associated with the elevation of inflammation in WAT.

### Improvement of insulin sensitivity in the HFD-fed *aP2-Cre/L-PGDS*^*flox/flox*^ mice

**Figure 7:**
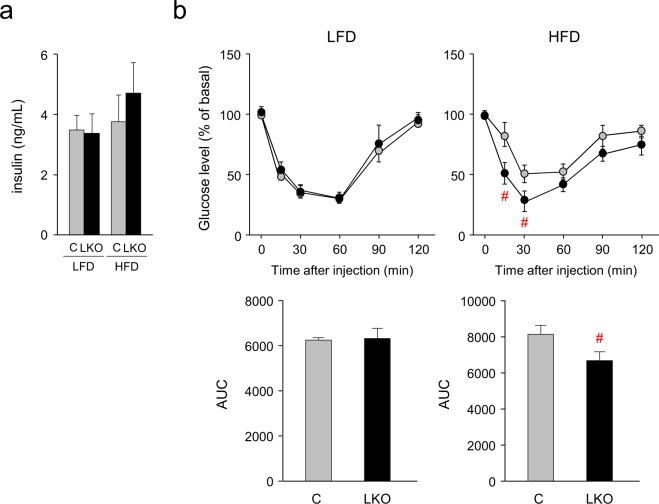
HFD-fed *aP2-Cre*/*L-PGDS* ^*flox/flox*^ mice exhibited increased insulin sensitivity. (**a**) Measurement of serum insulin level. Control *L-PGDS* ^*flox/flox*^ (C: *gray columns*) and *aP2-Cre*/*L-PGDS* ^*flox/flox*^ (LKO: *black columns*) mice (7-week-old; n = 5) were fed LFD or HFD for 11 weeks. Data are shown as means ± S.D. (**b**) Insulin tolerance test. Control *L-PGDS* ^*flox/flox*^ (*gray circles*; n = 5) and *aP2-Cre*/*L-PGDS* ^*flox/flox*^ (*black circles*; n = 5) mice (7-week-old) were fed LFD or HFD for 11 weeks, and fasted for 16 h before receiving an *i.p*. injection of 0.75 U/kg insulin. Serum glucose concentrations were measured at indicated time points. Data are shown as means ± S.D. The area under the curve (AUC) was compared by student’s *t*-test. ^#^*p* < 0.01, as compared with the HFD-fed *L-PGDS* ^*flox/flox*^ mice.

Next, we investigated insulin sensitivity in the LFD- and HFD-fed *aP2-Cre/L-PGDS* ^*flox/flox*^ and control *L-PGDS* ^*flox/flox*^ mice. Serum insulin levels were not significantly different between *aP2-Cre/L-PGDS* ^*flox/flox*^ and control *L-PGDS* ^*flox/flox*^ mice under both LFD and HFD conditions (Fig. 7a). However, serum insulin levels in the HFD-fed *aP2-Cre/L-PGDS* ^*flox/flox*^ mice were slightly higher than those of the *L-PGDS* ^*flox/flox*^ mice (Fig. 7a).

An intraperitoneal insulin tolerance test was carried out to elucidate insulin sensitivity of the LFD- and HFD-fed *aP2-Cre/L-PGDS* ^*flox/flox*^ and *L-PGDS* ^*flox/flox*^ mice. After injection of insulin, glucose levels in the *aP2-Cre/L-PGDS* ^*flox/flox*^ and *L-PGDS* ^*flox/flox*^ mice were similar under LFD conditions (Fig. 7b). In contrast, when fed an HFD, serum glucose levels in the *aP2-Cre/L-PGDS* ^*flox/flox*^ mice were lower than those of the *L-PGDS* ^*flox/flox*^ mice (Fig. 7b). These results reveal that adipose L-PGDS is associated with the impairment of insulin sensitivity in mice.

## Discussion

L-PGDS is widely expressed in various mouse tissues (Fig. 1a) and likely plays many different types of physiological and pathological functions^33^. L-PGDS is a bifunctional protein: one is to act as a PGD_2_-producing enzyme that catalyzes the isomerization of PGH_2_ to produce PGD_2_^34^, and the other is as a carrier protein for small lipophilic molecules such as retinal and retinoic acid^35^, biliverdin^36^ and bilirubin^37^, and gangliosides^38^. In adipocytes, PGD_2_ and its metabolites, Δ^12^-PGJ_2_ and 15-deoxy-Δ^12,14^-PGJ_2_, accelerate lipid accumulation through the DP2 receptors^28^ and PPARγ^12–14^, respectively. As shown in Fig. 4a, the PGD_2_ content is decreased in WAT of the *aP2-Cre*/*L-PGDS* ^*flox/flox*^ mice to about 50% of the *L-PGDS* ^*flox/flox*^ mice. Moreover, AT-56, an L-PGDS inhibitor, suppresses adipogenesis in mouse 3T3-L1 cells^14^. These results, taken together, indicate that L-PGDS acts as a PGD_2_-producing enzyme in adipocytes. The other half of PGD_2_ in WAT is considered to be produced by L-PGDS in non-adipocytes, such as endothelial cells^39^ of the blood vessels, or by H-PGDS in mast cells and other inflammatory cells^25^ within WAT.

Under HFD conditions, WAT was the third most enriched organ for L-PGDS mRNA expression followed by the brain and heart (Fig. 1a), and was the largest organ in the body. Thus, WAT is the most active organ in the total amount of L-PGDS gene expression under HFD conditions. The roles of L-PGDS in obesity have been identified by several *in vivo* studies^29–32^. PGD_2_-overproducing mice become obese under HFD conditions^29^. L-PGDS gene KO mice showed glucose intolerance and insulin resistance, and increased fat mass in the aorta under HFD conditions^30^. L-PGDS-ablated mice showed an improvement in glucose tolerance under HFD conditions^31^. In contrast, glucose intolerance or insulin resistance was not observed in the L-PGDS KO mice, but body weight gain and atherosclerotic lesions were increased in the aorta^32^. The roles of L-PGDS and/or PGD_2_ in obesity are controversial, because L-PGDS and PGD_2_ carry various functions in the body. Therefore, ablation of the L-PGDS gene or overproduction of PGD_2_ in the whole body may not be suitable for the evaluation of their roles in peripheral adipose tissue. Therefore, we employed the adipose-specific L-PGDS KO mice through the Cre-loxP system to find the functions of adipose L-PGDS and PGD_2_, and finally demonstrated that L-PGDS-produced PGD_2_ in premature adipocytes regulates body weight gain and insulin resistance under HFD conditions.

In this study, we used two distinct types of Cre-expressing mice under the control of adipocyte-specific promoters to generate the adipose-specific KO mice, *aP2-Cre*/*L-PGDS* ^*flox/flox*^ and *AdipoQ-Cre/L-PGDS* ^*flox/flox*^ mice. Between these two adipocyte-specific conditional KO mice, the *aP2-Cre*/*L-PGDS* ^*flox/flox*^ mice showed HFD-induced depletion of L-PGDS in adipocytes, whereas the *AdipoQ-Cre/L-PGDS* ^*flox/flox*^ mice did not show such a phenotype. When we examined the time course of L-PGDS expression in 3T3-L1 cells during their development from fibroblasts to adipocytes, the L-PGDS expression was similar to that of aP2 and earlier than that of AdipoQ (Fig. 1b). These results are supported by previous reports that showed the expression of the AdipoQ mRNA occurred slightly later than that of the aP2 mRNA in 3T3-F442A and 3T3-L1 cells^40^. Thus, L-PGDS and aP2 are expressed even in preadipocytes but AdipoQ was only expressed in mature adipocytes. Therefore, the *aP2-Cre*/*L-PGDS* ^*flox/flox*^ mice may be useful to delete L-PGDS under HFD conditions. In fact, we succeeded to disrupt the L-PGDS gene in adipose tissue under HFD conditions only by the use of the aP2 promoter-driven Cre, but not, of the AdipoQ promoter-driven one (Fig. 2b,c).

The HFD-fed *aP2-Cre*/*L-PGDS* ^*flox/flox*^ mice showed decreased body weight gain with the reduction of fat mass (Fig. 3a,b). The WAT of the *aP2-Cre*/*L-PGDS* ^*flox/flox*^ mice were smaller in size than the control *L-PGDS* ^*flox/flox*^ mice (Fig. 4d,e), suggesting that adipose L-PGDS and PGD_2_ are associated with the enhancement of obesity, together with the enlargement of adipose cells. However, the loss of the L-PGDS gene in WAT and decreased body weight gain were not observed in the HFD-fed *AdipoQ-Cre/L-PGDS* ^*flox/flox*^ mice (Fig. 3a,b), although the Cre transgene was expressed in WAT in these mice (Fig. 2b). Further expression profile analysis demonstrated that induction of the expression of the AdipoQ gene came later than those of the L-PGDS and aP2 genes in mouse adipocytic 3T3-L1 cells (Fig. 1b). These results suggest that the delayed induction of AdipoQ gene expression did not disrupt the L-PGDS gene in WAT of the HFD-fed *AdipoQ-Cre/L-PGDS* ^*flox/flox*^ mice and that L-PGDS in WAT had already been produced in premature adipocytes. Mature adipocytes with active gene expression of AdipoQ did not induce L-PGDS by the HFD feeding and were not mainly involved in L-PGDS-mediated increases in body weight and fat mass caused by HFD. L-PGDS in mature adipocytes may be involved in other functions, such as the transport of various lipophilic ligands.

The aP2 gene is also expressed in macrophages^3,41^, liver, and brain. However, the expression level of the aP2 gene in macrophages is about 10^−4^-fold lower than that in adipocytes^3^. In the HFD-fed *aP2-Cre/L-PGDS* ^*flox/flox*^ mice, the transcription level of the Cre transgene was negligible in brain and liver. (Fig. 2b). The expression of L-PGDS was not affected by HFD in those organs of the *aP2-Cre/L-PGDS* ^*flox/flox*^ mice (Fig. 2b,c). The expression level of the L-PGDS gene was very low in the stromal vascular fraction (SVF) of obese adipose tissue and peritoneal macrophages prepared from LFD- and HFD-fed wild type mice (data not shown). All of these results, taken together, indicate that the HFD-induced upregulation of L-PGDS occurs not in macrophages, but predominantly in adipocytes. In this study, we have not yet identified the cells that express L-PGDS in obese adipose tissue. As SVF consists of a heterogeneous population that includes endothelial cells, erythrocytes, fibroblasts, and lymphocytes as well as pre-adipocytes, and adipocyte progenitor cells, we will undertake further analyses using pure SVF to identify the L-PGDS-expressing cells in obese adipose tissue. Moreover, we will investigate the function of L-PGDS in adipose macrophages by using macrophage-specific L-PGDS gene KO mice, *lysozyme M* (*LysM*)*-Cre/L-PGDS* ^*flox/flox*^ mice.

Another important finding in this study was that the adipose-specific disruption of the L-PGDS gene showed an anti-inflammatory effect. In obese adipose tissue, at least two different macrophages, M1 and M2, are found^42^. M1 macrophages make up the majority of adipose macrophages that exist in WAT of obese^42^. However, it is still unclear whether obesity induces the recruitment of monocytes that become M1 macrophages, or if HFD changes the phenotype of the tissue contianing M2 macrophages. In WAT of the HFD-fed *aP2-Cre*/*L-PGDS* ^*flox/flox*^ mice, the expression levels of the M1 macrophage marker genes were all decreased (Fig. 6 and Supplementary Fig. S4), whereas the transcription levels of the M2 macrophage marker genes were either enhanced or not altered under HFD conditions (Fig. 6 and Supplementary Fig. S4). The loss of adipose L-PGDS during obesity prevents HFD-induced inflammation. Obesity and insulin resistance are closely associated with inflammation in adipose tissue^43–45^. Accelerated *de novo* adipogenesis and lipogenesis with repressed lipolysis are closely associated with insulin sensitivity^3^. M1 macrophages in adipose tissue produce pro-inflammatory cytokines such as TNFα, which induces insulin resistance and suppresses the expression of PPARγ^3^. PGD_2_ may be involved in enhancing inflammation in WAT of the HFD-fed mice. The HFD-fed *aP2-Cre*/*L-PGDS* ^*flox/flox*^ mice showed improved insulin sensitivity (Fig. 7b) with lowered expression of TNFα in WAT (Fig. 6 and Supplementary Fig. S4). In a previous study, when COX activity was inhibited by indomethacin in the HFD-fed mice, insulin resistance was prevented by the decreased plasma PGD_2_ level and reduced expression of the macrophage marker genes in adipose tissue^46^. Thus, the absence of adipose L-PGDS and PGD_2_ may prevent the phenotypic pro-inflammatory state that is induced under HFD. Macrophages express H-PGDS and infiltrate into the enlarged adipose tissue^26^. Thus, PGD_2_ may be produced by H-PGDS in macrophages that have infiltrated the enlarged adipose tissue. The roles of H-PGDS-produced PGD_2_ in macrophages that have infiltrated obese adipose tissue should be further elucidated.

**Figure 8:**
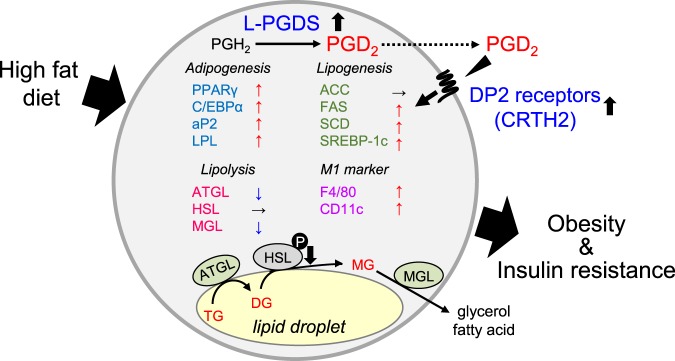
Summary of activation of obesity by L-PGDS-produced PGD_2_ through DP2 receptors in adipocytes under HFD conditions. DG: diacylglyceride, MG: monoacylglyceride. M1 marker means M1 macrophage marker. *Arrowheads* indicate the changes of the expression levels under HFD conditions.

As summarized in Fig. 8, adipose L-PGDS enhances body weight gain with the elevation of fat mass under HFD conditions. Adipose-specific disruption of the L-PGDS gene in the *aP2-Cre*/*L-PGDS* ^*flox/flox*^ mice under HFD shows an improvement in insulin sensitivity. The molecular mechanism for the decrease of adiposity in the HFD-fed *aP2-Cre*/*L-PGDS* ^*flox/flox*^ mice is still unclear. An *in vitro* study demonstrated that PGD_2_ suppressed the lipolysis through the DP2 receptors in adipocytes^28^. Thus, adipocyte PGD_2_ might be related to the regulation of lipolysis *in vivo*. Further *in vivo* studies are needed to elucidate the whole molecular mechanism of PGD_2_-regulated adiposity. In this study, we conclude that adipocyte-specific inhibition of L-PGDS or the DP2 receptors is potentially useful for the treatment of obesity and obesity-mediated insulin resistance.

## Methods

### Animals

*L-PGDS* ^*flox/flox*^ mice (C57BL/6 background) carrying a conditional L-PGDS deleted allele in which exon 2 to 7 including whole open reading frame of L-PGDS are flanked by two loxP sites. *aP2-Cre* and *AdipoQ-Cre* mice (C57BL/6 background; Jackson Laboratory, Bar Harbor, ME, USA) carry the Cre transgene driven by the aP2 promoter/enhancer or AdipoQ promoter, respectively, both of which are predominantly expressed in adipocytes^47,48^. The *aP2-Cre*/*L-PGDS* ^*flox/flox*^ or *AdipoQ-Cre*/*L-PGDS* ^*flox/flox*^ mice were generated by intercrossing mice bearing a conditional L-PGDS allele (*L-PGDS* ^*flox/flox*^) with *aP2-Cre*/*L-PGDS* ^*flox/flox*^ or *AdipoQ-Cre*/*L-PGDS* ^*flox/flox*^. Littermates lacking the aP2-Cre or AdipoQ-Cre transgene (*L-PGDS* ^*flox/flox*^) were used as the control. In the present study, we used only male mice to exclude the effects of female hormonal imbalance.

Mice were maintained with a 12-h light/12-h dark photoperiod in a humidity- and temperature-controlled room (55% at 24 °C). Water and food were available *ad libitum*. The animals were fed either LFD (FR-2, 4.8% fat; Funabashi Farm, Chiba, Japan) or HFD (35% fat; Research Diets, New Brunswick, NJ, USA).

The animal study was approved by the Animal committee of Osaka University of Pharmaceutical Sciences. Animals were handled in accordance with the principles and guidelines established by the respective committee. Every effort was made to minimize the number of animals used in these studies and their suffering.

### Cell culture

Mouse adipocytic 3T3-L1 cells (Human Science Research Resources Bank, Osaka, Japan) were grown in Dulbecco’s modified eagle medium (DMEM; Sigma, St. Louis, MO, USA) containing 10%(v/v) fetal bovine serum (CORNING, Corning, NY, USA) and antibiotics (Nacalai Tesque, Kyoto, Japan) at 37 °C in a humidified atmosphere of 5% CO_2_. For adipocyte differentiation, 3T3-L1 cells were cultured in DMEM containing insulin (10 μg/ml; Sigma), 1 μM dexamethasone (Sigma), and 0.5 mM 3-isobutyl-1-methylxanthine (Nacalai Tesque). On day 2, medium was exchanged to DMEM containing insulin (10 μg/ml) alone and subsequently changed every 2 days.

### Measurement of RNA level

Extraction of RNA and synthesis of first-strand cDNAs were performed as described previously^49^. Measurement of the mRNA levels by qPCR was conducted using the LightCycler System (Roche Diagnostics, Mannheim, Germany) and ABI 7500 Real-Time PCR System (Thermo Fischer Scientific, Waltham, MA, USA) with THUNDERBIRD SYBR qPCR Mix (Toyobo, Osaka, Japan) and Power SYBR Green PCR Master Mix (Thermo Fischer Scientific), and primers (Supplemental Table S1). Transcription level of the desired gene was normalized to that of TATA-binding protein (TBP) as the internal control.

### Western blot analysis

Proteins from tissues were prepared as follows. Tissues were disrupted in RIPA buffer {50 mM Tris-Cl (pH 8.0), 150 mM NaCl, 0.5%(w/v) sodium deoxycholate, 0.1%(v/v) SDS, 1% (v/v) NP-40}containing 1%(v/v) Triton X-100 and a protease inhibitor cocktail (Nacalai Tesque) by a Bead beater-type homogenizer (TAITEC, Saitama, Japan). After centrifugation to remove debris, protein concentrations of the supernatants (crude extracts) were determined by using a Pierce BCA Protein Assay Kit (Thermo Fisher Scientific). Proteins were separated by SDS-PAGE, followed by blotting onto the PVDF membranes (Immobilon; Merck, Kenilworth, NJ, USA). Further analysis by Western blotting was carried out as described previously^50^. Mouse L-PGDS polyclonal antibody and β-actin monoclonal antibody (Sigma), and anti-mouse or anti-rabbit IgG antibody conjugated with horseradish peroxidase (Santa Cruz Biotech., Dallas, TX, USA) were used in this study.

### Enzyme immunoassay (EIA)

The PGs in WAT were extracted as described previously^29^. Production of PGD_2_ was measured by using a PGD_2_ MOX EIA Kit (Cayman Chemical, Ann Arbor, MI, USA) according to the manufacturer’s instructions.

### Computed tomography (CT)

Mice were anesthetized with Nembutal (50 mg/kg of body weight, *i.p*.; Abbott Laboratories, North Chicago, IL, USA). CT analysis was carried out by a micro-CT scanner (LaTheta LCT-100; Hitachi, Tokyo, Japan). The analysis of CT data was carried out by the use of LaTheta software (Hitachi). The vWAT and sWAT, and muscle weights were measured from images at the level of the umbilicus. Subcutaneous WAT was defined as the extraperitoneal fat between skin and muscle. The intraperitoneal part with the same density as the subcutaneous fat layer was defined as vWAT. Proportions of vWAT and sWAT were determined by automatic planimetry as described previously^29^.

### Histological analysis

Tissues were fixed in 4%(v/v) paraformaldehyde and embedded in Tissue-Tek O.C.T. Compound (Sakura Finetek, Torrance, CA, USA). Frozen sections (10 μm-thickness) were stained with hematoxylin and eosin. The sections were observed using an ECLIPSE E600 microscope (Nikon, Tokyo, Japan). At least three discontinuous sections were used for evaluation.

### Serum biochemical parameter

Mice were fasted for 16 h prior to the collection of blood samples. Blood was collected from the abdominal aorta. Serum TG levels were determined by using Triglyceride Test Wako (Wako Pure Chemical, Osaka Japan), and insulin levels were measured by using ELISA kits (SHIBAYAGI, Gunma, Japan), according to the manufacturer’s instructions. Serum TG, NEFA, total cholesterol, low-density lipoprotein (LDL), and high-density lipoprotein (HDL) levels were determined by using L-Type TG M test, NEFA-C, Cholesterol M, L-Type LDL-C, and L-Type HDL-C Kits (Wako Pure Chemical) according to the manufacturer’s instructions.

### Insulin tolerance test

Mice were fasted for 16 h prior to intraperitoneal (*i.p*.) injection of insulin (0.75 IU/kg of body weight; HUMULIN^®^; Eli Lilly, Indianapolis, IN, USA). Blood was collected from the tail vein and glucose levels were immediately measured by the use of a MEDISAFE MINI Blood Glucose Monitoring System (Terumo, Tokyo, Japan). Blood glucose levels were measured at 0, 15, 30, 60, 90, and 120 min after injection of insulin.

### Statistical analysis

Data are presented as the means ± S.E. or S.D. Statistical significance was determined by using the paired Student’s *t* test. For comparison of more than two groups with comparable variances, one-way ANOVA and Tukey’s *post-hoc* test were carried out. *p* < 0.05 was considered significant.

## Supplementary information

Supplemental Information

## Data Availability

The data generated and analyzed during this study that are not included in the published article are available from the corresponding author upon request.

## References

[1] Friedman JM (2004). Modern science versus the stigma of obesity. Nat. Med..

[2] Cornier MA (2008). The metabolic syndrome. Endocr. Rev..

[3] Guilherme A, Virbasius JV, Puri V, Czech MP (2008). Adipocyte dysfunctions linking obesity to insulin resistance and type 2 diabetes. Nat. Rev. Mol. Cell Biol..

[4] Spiegelman BM, Flier JS (2001). Obesity and the regulation of energy balance. Cell.

[5] Galic S, Oakhill JS, Steinberg GR (2010). Adipose tissue as an endocrine organ. Mol. Cell. Endocrinol..

[6] Lefterova MI, Lazar MA (2009). New developments in adipogenesis. Trends Endocrinol. Metab..

[7] White UA, Stephens JM (2010). Transcriptional factors that promote formation of white adipose tissue. Mol. Cell. Endocrinol..

[8] Rosen E, Eguchi J, Xu Z (2009). Transcriptional targets in adipocyte biology. Expert. Opin. Ther. Targets.

[9] Masoodi M, Kuda O, Rossmeisl M, Flachs P, Kopecky J (2015). Lipid signaling in adipose tissue: Connecting inflammation & metabolism. Biochim. Biophys. Acta.

[10] Fujimori K (2012). Prostaglandins as PPARγ modulators in adipogenesis. PPAR Res..

[11] Fujimori K, Aritake K, Urade Y (2007). A novel pathway to enhance adipocyte differentiation of 3T3-L1 cells by up-regulation of lipocalin-type prostaglandin D synthase mediated by liver X receptor-activated sterol regulatory element-binding protein-1c. J. Biol. Chem..

[12] Forman BM (1995). 15-Deoxy-Δ^12,14^-prostaglandin J_2_ is a ligand for the adipocyte determination factor PPARγ. Cell.

[13] Kliewer SA (1995). A prostaglandin J_2_ metabolite binds peroxisome proliferator-activated receptor γand promotes adipocyte differentiation. Cell.

[14] Fujimori K, Maruyama T, Kamauchi S, Urade Y (2012). Activation of adipogenesis by lipocalin-type prostaglandin D synthase-generated Δ^12^-PGJ_2_ acting through PPARγ-dependent and independent pathways. Gene.

[15] Fujimori K, Yano M, Ueno T (2012). Synergistic suppression of early phase of adipogenesis by microsomal PGE synthase-1 (PTGES1)-produced PGE_2_ and aldo-keto reductase 1B3-produced PGF_2α_. PLoS One.

[16] Tsuboi H, Sugimoto Y, Kainoh T, Ichikawa A (2004). Prostanoid EP4 receptor is involved in suppression of 3T3-L1 adipocyte differentiation. Biochem. Biophys. Res. Commun..

[17] Inazumi T (2011). Prostaglandin E-EP4 signaling suppresses adipocyte differentiation in mouse embryonic fibroblasts via an autocrine mechanism. J. Lipid Res..

[18] Fujimori K, Yano M, Miyake H, Kimura H (2014). Termination mechanism of CREB-dependent activation of COX-2 expression in early phase of adipogenesis. Mol. Cell. Endocrinol..

[19] Fujimori K (2010). Suppression of adipocyte differentiation by aldo-keto reductase 1B3 acting as prostaglandin F_2α_ synthase. J. Biol. Chem..

[20] Tirard J (2007). A novel inhibitory protein in adipose tissue, the aldo-keto reductase AKR1B7: its role in adipogenesis. Endocrinology.

[21] Casimir DA, Miller CW, Ntambi JM (1996). Preadipocyte differentiation blocked by prostaglandin stimulation of prostanoid FP2 receptor in murine 3T3-L1 cells. Differentiation.

[22] Miller CW, Casimir DA, Ntambi JM (1996). The mechanism of inhibition of 3T3-L1 preadipocyte differentiation by prostaglandin F_2α_. Endocrinology.

[23] Ueno T, Fujimori K (2011). Novel suppression mechanism operating in early phase of adipogenesis by positive feedback loop for enhancement of cyclooxygenase-2 expression through prostaglandin F_2α_ receptor mediated activation of MEK/ERK-CREB cascade. Febs J..

[24] Urade Y, Eguchi N (2002). Lipocalin-type and hematopoietic prostaglandin D synthases as a novel example of functional convergence. Prostaglandins Other Lipid Mediat..

[25] Kanaoka Y, Urade Y (2003). Hematopoietic prostaglandin D synthase. Prostaglandins, Leukot. Essent. Fatty Acids.

[26] Redensek A (2011). Expression and detrimental role of hematopoietic prostaglandin D synthase in spinal cord contusion injury. Glia.

[27] Smith WL, Urade Y, Jakobsson PJ (2011). Enzymes of the cyclooxygenase pathways of prostanoid biosynthesis. Chem. Rev..

[28] Wakai E, Aritake K, Urade Y, Fujimori K (2017). Prostaglandin D_2_ enhances lipid accumulation through suppression of lipolysis via DP2 (CRTH2) receptors in adipocytes. Biochem. Biophys. Res. Commun..

[29] Fujitani Y (2010). Pronounced adipogenesis and increased insulin sensitivity caused by overproduction of prostaglandin D_2_*in vivo*. FEBS J..

[30] Ragolia L (2005). Accelerated glucose intolerance, nephropathy, and atherosclerosis in prostaglandin D_2_ synthase knock-out mice. J. Biol. Chem..

[31] Virtue S (2012). A new role for lipocalin prostaglandin D synthase in the regulation of brown adipose tissue substrate utilization. Diabetes.

[32] Tanaka R (2009). Knockout of the l-pgds gene aggravates obesity and atherosclerosis in mice. Biochem. Biophys. Res. Commun..

[33] Urade Y, Hayaishi O (2000). Biochemical, structural, genetic, physiological, and pathophysiological features of lipocalin-type prostaglandin D synthase. Biochim. Biophys. Acta.

[34] Urade Y, Fujimoto N, Hayaishi O (1985). Purification and characterization of rat brain prostaglandin D synthetase. J. Biol. Chem..

[35] Tanaka T (1997). Lipocalin-type prostaglandin D synthase (beta-trace) is a newly recognized type of retinoid transporter. J. Biol. Chem..

[36] Inui T (2014). Lipocalin-type prostaglandin D synthase scavenges biliverdin in the cerebrospinal fluid of patients with aneurysmal subarachnoid hemorrhage. J. Cereb. Blood Flow Metab..

[37] Beuckmann CT (1999). Binding of biliverdin, bilirubin, and thyroid hormones to lipocalin-type prostaglandin D synthase. Biochemistry.

[38] Mohri I (2006). Lipocalin-type prostaglandin D synthase is up-regulated in oligodendrocytes in lysosomal storage diseases and binds gangliosides. J. Neurochem..

[39] Negoro H (2005). Endogenous prostaglandin D_2_ synthesis decreases vascular cell adhesion molecule-1 expression in human umbilical vein endothelial cells. Life Sci.

[40] Hu E, Liang P, Spiegelman BM (1996). AdipoQ is a novel adipose-specific gene dysregulated in obesity. J Biol Chem.

[41] Kang S, Kong X, Rosen ED (2014). Adipocyte-specific transgenic and knockout models. Methods Enzymol..

[42] Castoldi AN, de Souza C, Camara NO, Moraes-Vieira PM (2015). The Macrophage Switch in ObesityDevelopment. Front. Immunol..

[43] Tateya S, Kim F, Tamori Y (2013). Recent advances in obesity-induced inflammation and insulin resistance. Front. Endocrinol..

[44] Vieira-Potter VJ (2014). Inflammation and macrophage modulation in adipose tissues. Cell Microbiol.

[45] Johnson AM, Olefsky JM (2013). The origins and drivers of insulin resistance. Cell.

[46] Fjaere E (2014). Indomethacin treatment prevents high fat diet-induced obesity and insulin resistance but not glucose intolerance in C57BL/6J mice. J. Biol. Chem..

[47] He W (2003). Adipose-specific peroxisome proliferator-activated receptor γ knockout causes insulin resistance in fat and liver but not in muscle. Proc. Natl. Acad. Sci. USA.

[48] Eguchi J (2011). Transcriptional control of adipose lipid handling by IRF4. Cell Metab..

[49] Yuyama M, Fujimori K (2014). Suppression of adipogenesis by valproic acid through repression of USF1-activated fatty acid synthesis in adipocytes. Biochem. J..

[50] Nagai, S., Matsumoto, C., Shibano, M. & Fujimori, K. Suppression of fatty acid and triglyceride synthesis by the flavonoid orientin through decrease of C/EBPδ expression and inhibition of PI3K/Akt-FOXO1 signaling in adipocytes. *Nutrients* **10**, 10.3390/nu10020130 (2018). 10.3390/nu10020130 PMC5852706 29373533

