# Supplementary material for: L-PGDS-produced PGD2 in premature, but not in mature, adipocytes increases obesity and insulin resistance
Source: Sci Rep. 2019 Feb 13;9:1931. doi: 10.1038/s41598-018-38453-y (PMC6374461; doi:10.1038/s41598-018-38453-y)
Supplement: Supplementary file 1 — Supplemental Information [file 41598_2018_38453_MOESM1_ESM.doc]

***Supplementary Information***

**L-PGDS-produced PGD_2_ in premature, but not in mature, adipocytes increases obesity and insulin resistance**

Ko Fujimori ^1^, Kosuke Aritake ^2^, Yo Oishi ^3^, Nanae Nagata ^4^, Toko Maehara ^1^, Michael Lazarus ^3^, and Yoshihiro Urade ^5,6^

^1^ Department of Pathobiochemistry, Osaka University of Pharmaceutical Sciences, 4-20-1 Nasahara, Takatsuki, Osaka 569-1094, Japan

^2^ Laboratory of Chemical Pharmacology, Daiichi University of Pharmacy, 22-1 Tamagawa-cho, Minami-ku, Fukuoka 815-8511, Japan

^3^ International Institute for Integrative Sleep Medicine (WPI-IIIS), University of Tsukuba, 1-1-1 Tennodai, Tsukuba, Ibaraki 305-8575 Japan

^4^ Department of Animal Radiology and Graduate School of Agriculture and Life Sciences, The University of Tokyo, 1-1-1 Yayoi, Bunkyo-ku, Tokyo 113-8657, Japan.

^5^ The University of Tokyo Hospital, 7-3-1 Hongo, Bunkyo-ku, Tokyo 113-8655, Japan

^6^ Graduate School of Pharmaceutical Sciences, Kitasato University, 5-9-1 Shirokane, Minato-ku, Tokyo 108-8641, Japan.

**Supplemental Materials and Methods**

**Genotyping**

Mouse tail-tip DNAs were prepared by the use of KAPA Mouse Genotyping Kit (KAPA Biosystems, Wilmington, MA, USA). For determination of *floxed* and *null* alleles, PCR was conducted by the use of primer set: FL-F (5’-**GGGTGTTCTGGGCACTGTCAG**-3’) and FL-R2 (5’-**CCTGGCTCCTTGGAGACCCCTGCTGC**-3’) yielding 2,513-bp and 513-bp products for *floxed* and *null* alleles, respectively. PCR conditions were: 30-35 cycles of 94 °C for 20 s, 55-60 °C for 20 s, and 74 °C for 20-120 s by using EmeraldAmp MAX PCR Master Mix (Takara Bio, Shiga, Japan) or GoTaq DNA Polymerase (Promega, Madison, WI, USA). The resultant PCR products were analyzed by an agarose gel electrophoresis.

**Supplementary Figure S1. Genotyping analysis of *floxed* and *null* L-PGDS alleles in various tissues.**

DNA was prepared from various tissues of the *aP2-Cre*/*L-PGDS^flox/flox^* and *L-PGDS^flox/flox^* mice (7-week-old) after 11 weeks LFD or HFD. Each amplification size was indicated by right-side of the pictures; *floxed* L-PGDS allele: 2,513-bp and *null* L-PGDS: 513-bp (FL-F and FL-R2 as the primers). B: brain, H: heart, Lu: lung, Li: liver, K: kidney, S: spleen, I: small intestine, M: skeletal muscle, W: WAT.

**Supplementary Figure S2. Comparison of the *L-PGDS^flox/flox^*mice and wild-type mice.**

a. Body weight change in the *L-PGDS^flox/flox^* and wild-type mice (WT; C57BL/6, 5-week-old; *n=*3) for 7 weeks under LFD or HFD conditions. Body weight was measured every week. Data are shown as means ± S.D. b. Expression of the L-PGDS gene in the HFD-fed wild-type (W; *white columns*) and *L-PGDS^flox/flox^* (flox; *gray columns*) mice. Mice (5-week-old; n=3) were fed an HFD for 7 weeks and the mRNA levels of the L-PGDS gene in the brain, liver, and vWAT were measured by qPCR. Data are shown as means ± S.D.

**Supplementary Figure S3. Change of gene expression levels in sWAT of the LFD- or HFD-fed mice**

a. Expression of the PG synthetic genes in sWAT of the LFD- or HFD-fed control *L-PGDS^flox/flox^* (C: *gray columns*) and *aP2-Cre/L-PGDS^flox/flox^* mice (L: *black columns*; 18-week-old; n=5-6) under LFD or HFD. The expression levels of the indicated genes were measured by qPCR. Data are present as means ± S.D. **p*<0.01, as indicated by the brackets. b. Expression of the adipogenic, lipogenic, and lipolytic genes in sWAT of the LFD- or HFD-fed control *L-PGDS^flox/flox^* (C: *gray columns*) and *aP2-Cre*/*L-PGDS^flox/flox^* mice (L: *black columns*; 18-week-old; n=5-6) under LFD or HFD. The expression levels of the indicated genes were measured by qPCR. Data are present as means ± S.D. **p*<0.01, ^#^*p*<0.01, as indicated by the brackets.

**Supplementary Figure S4. Expression of the inflammatory genes in sWAT of the LFD- or HFD-fed mice**

Expression of the macrophage (M1 and M2) marker genes in sWAT of the LFD- or HFD-fed control *L-PGDS^flox/flox^* (C: *gray columns*) and *aP2-Cre*/*L-PGDS^flox/flox^* mice (L: *black columns*; 18-week-old; n=5-6) under LFD or HFD. The expression levels of the indicated genes were measured by qPCR. Data are present as means ± S.D. **p*<0.01, ^#^*p*<0.01, as indicated by the brackets.

**Supplemental Table S1. Nucleotide sequences of primers used in qPCR**

gene accession No. 　 forward primer reverse primer

PPARγ NM_011146 5'-CAAGAATACCAAAGTGCGATCAA-3’ 5’-GAGCTGGGTCTTTTCAGAATAATAAG-3’

C/EBPα NM_007678 5’-CTGGAAAGAAGGCCACCTC-3’ 5’-AAGAGAAGGAAGCGGTCCA-3’

aP2 NM_024406 5’-CAGCCTTTCTCACCTGGAAG-3’ 5’-TTGTGGCAAAGCCCACTC-3’

AdipoQ NM_009605 5’-CAGGCATCCCAGGACATC-3’ 5’-TCTCACCCTTAGGACCAAGAAG-3’

LPL NM_008509 5’-CTCGCTCTCAGATGCCCTAC-3’ 5’-AGGCCTGGTTGTGTTGCTT-3’

ACC NM_133360 5’-GCGTCGGGTAGATCCAGTT-3’　　　　 5’-CTCAGTGGGGCTTAGCTCTG-3’

FAS NM_007988 5’-GTTGGGGGTGTCTTCAACC-3’ 5’-GAAGAGCTCTGGGGTCTGG-3’

SCD NM_009127 5’-CGTCTGGAGGAACATCATTCT-3’ 5’-CAGAGCGCTGGTCATGTAGT-3’

SREBP-1 NM_011480 5’-GGAGCCATGGATTGCACATT-3’ 5’-GCTTCCAGAGAGGAGGCCAG-3’

ATGL NM_001163689 5’-TGACCATCTGCCTTCCAGA-3’ 5’-TGTAGGTGGCGCAAGACA-3’

HSL NM_010719 5’-GCACTGTGACCTGCTTGGT-3’ 5’-CTGGCACCCTCACTCCATA-3’

MGL NM_011844 5’-TCGGAACAAGTCGGAGGT-3’ 5’-TCAGCAGCTGTATGCCAAAG-3’

F4/80 NM_010130 5’-GGAGGACTTCTCCAAGCCTATT-3’ 5’-AGGCCTCTCAGACTTCTGCTT-3’

CD11c NM_021334 5’-AGCCTCAAGACAGGACATCG-3’ 5’-TGAATCCTGGAGGGGATCT-3’

CD163 NM_001170395 5’-TCTCAGTGCCTCTGCTGTCA-3’ 5’-CGCCAGTCTCAGTTCCTTCT-3’

CD204 NM_001113326 5’-GTCTTCTTTACCAGCAATGACAAA-3’ 5’-CAGTCAGCATCCTCTTGTTCA-3’

CD206 NM_008625 5’-CCACAGCATTGAGGAGTTTG-3’ 5’-ACAGCTCATCATTTGGCTCA-3’

TBP NM_013684 5’-GTGATGTGAAGTTCCCCATAAGG-3’ 5’-CTACTGAACTGCTGGTGGGTCA-3’
